# An update on *Acanthamoeba* keratitis: diagnosis, pathogenesis and treatment

**DOI:** 10.1051/parasite/2015010

**Published:** 2015-02-18

**Authors:** Jacob Lorenzo-Morales, Naveed A. Khan, Julia Walochnik

**Affiliations:** 1 University Institute of Tropical Diseases and Public Health of the Canary Islands, University of La Laguna, Avda. Astrofísico Fco. Sánchez, S/N 38203 La Laguna, Tenerife, Canary Islands Spain; 2 Department of Biological and Biomedical Sciences, Aga Khan University Karachi Pakistan; 3 Institute of Specific Prophylaxis and Tropical Medicine, Medical University of Vienna Vienna Austria

**Keywords:** *Acanthamoeba*, keratitis, diagnosis, therapy, pathogenesis

## Abstract

Free-living amoebae of the genus *Acanthamoeba* are causal agents of a severe sight-threatening infection of the cornea known as *Acanthamoeba* keratitis. Moreover, the number of reported cases worldwide is increasing year after year, mostly in contact lens wearers, although cases have also been reported in non-contact lens wearers. Interestingly, *Acanthamoeba* keratitis has remained significant, despite our advances in antimicrobial chemotherapy and supportive care. In part, this is due to an incomplete understanding of the pathogenesis and pathophysiology of the disease, diagnostic delays and problems associated with chemotherapeutic interventions. In view of the devastating nature of this disease, here we present our current understanding of *Acanthamoeba* keratitis and molecular mechanisms associated with the disease, as well as virulence traits of *Acanthamoeba* that may be potential targets for improved diagnosis, therapeutic interventions and/or for the development of preventative measures. Novel molecular approaches such as proteomics, RNAi and a consensus in the diagnostic approaches for a suspected case of *Acanthamoeba* keratitis are proposed and reviewed based on data which have been compiled after years of working on this amoebic organism using many different techniques and listening to many experts in this field at conferences, workshops and international meetings. Altogether, this review may serve as the milestone for developing an effective solution for the prevention, control and treatment of *Acanthamoeba* infections.

## Abstract (fr)

Les amibes à vie libre du genre *Acanthamoeba* sont les agents causant une infection sévère de la cornée, dangereuse pour la vue, appelée kératite à *Acanthamoeba*. De plus, le nombre de cas signalés à travers le monde est en augmentation année après année, principalement chez les porteurs de lentilles de contact, bien que des cas de kératite à *Acanthamoeba* aient également été signalés chez les non-porteurs de lentilles. Fait intéressant, la kératite à *Acanthamoeba* est restée significative, en dépit de nos progrès dans la chimiothérapie antimicrobienne et les soins de soutien. En partie, cela est dû à une compréhension incomplète de la pathogenèse et la physiopathologie de la maladie, aux retards du diagnostic et aux problèmes associés aux interventions chimiothérapeutiques. Compte tenu de la nature dévastatrice de cette maladie, nous présentons ici notre compréhension actuelle de la kératite à *Acanthamoeba* et des mécanismes moléculaires associés à la maladie, ainsi que les traits de virulence de *Acanthamoeba* qui peuvent être des cibles potentielles pour l’amélioration du diagnostic, les interventions thérapeutiques et/ou pour l’élaboration de mesures préventives. Des approches moléculaires comme la protéomique, l’ARNi et des approches consensuelles de diagnostic pour un cas suspecté de kératite à *Acanthamoeba* sont proposées et examinées sur la base des données qui ont été compilées après des années de travail sur cet organisme amibien, utilisant de nombreuses techniques différentes et l’écoute de nombreux experts sur ce domaine à des conférences, ateliers et réunions internationales. Au total, cette étude peut servir de jalon pour développer une solution efficace pour la prévention, le contrôle et le traitement des infections à *Acanthamoeba*.

## 1. Introduction – What is *Acanthamoeba* keratitis?

**Figure 1.**
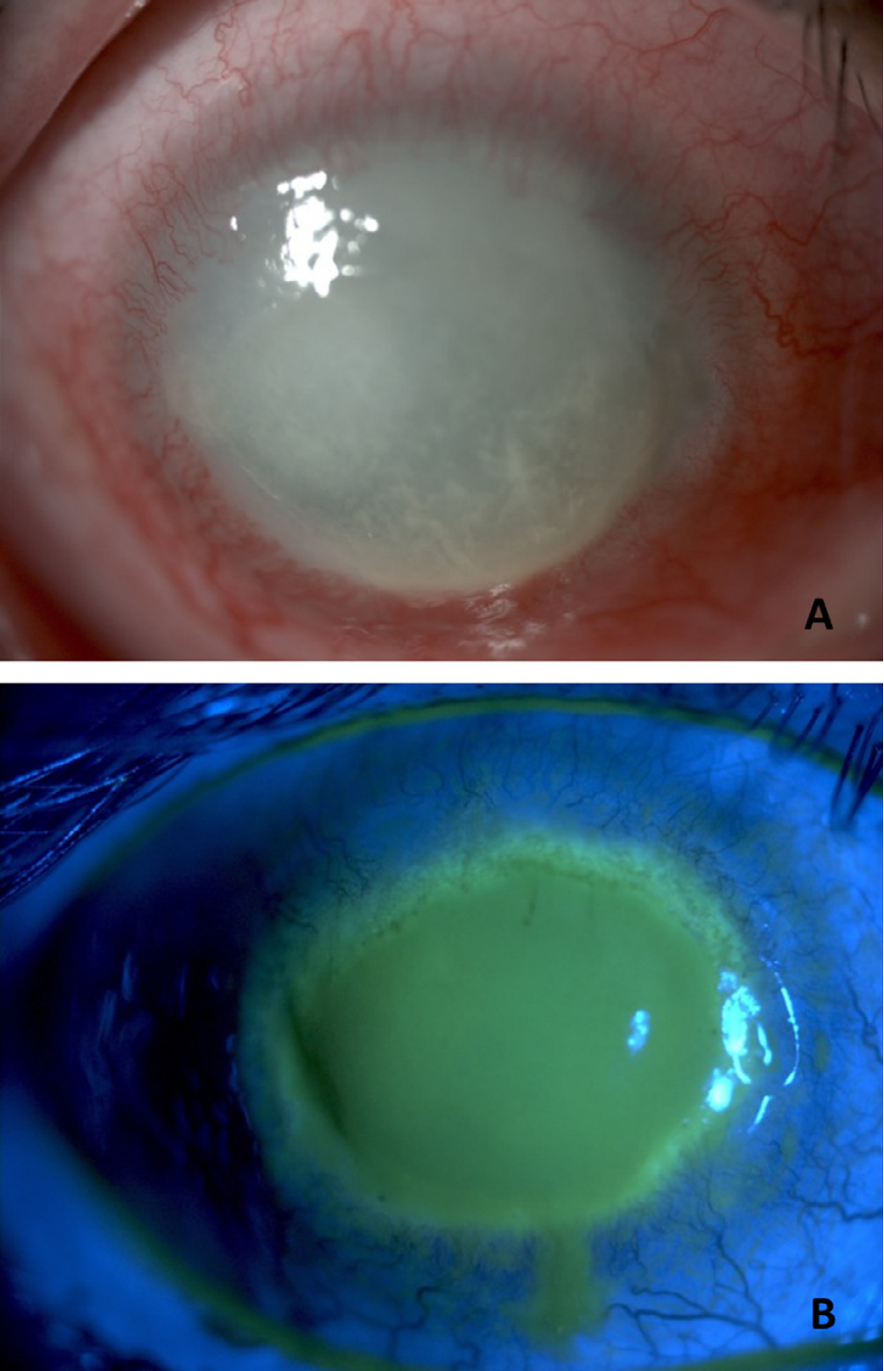
(A) Corneal melting and vascularization in a patient with *Acanthamoeba* keratitis. (B) Observed corneal damage in AK is shown after sodium fluorescein application. Original.

*Acanthamoeba* species are the causative agents of a sight-threatening infection of the cornea known as *Acanthamoeba* keratitis (AK) (Fig. 1). Interestingly, AK is increasingly being recognized as a severe sight-threatening ocular infection, worldwide. Although contact lens (CL) wear is the leading risk factor for AK, *Acanthamoeba* spp. can cause infection in non-contact lens wearers. Patients with AK may experience pain with photophobia, ring-like stromal infiltrate, epithelial defect and lid oedema. If AK is not treated adequately and aggressively, it can lead to loss of vision [18, 46, 47, 56, 87, 111, 112, 117].

Diagnosis of AK is challenging, and the available treatments are lengthy and not fully effective against all strains. Moreover, the pathogenesis of *Acanthamoeba* keratitis is still under study, and the identification of the key factors involved in this process should be useful for the development of fully effective therapies. The current difficulty in effective treatment is due to the resistant cyst stage of *Acanthamoeba*. Together with common misdiagnosis of AK in most cases and a lack of a consensus for AK diagnosis, AK has remained significant. Nevertheless, AK is still considered a rare disease and is included in the Orphanet database (ORPHA67043) and with an estimated prevalence of 1–9/100,000.

## 2. Diagnostics of AK

The most important step in AK diagnosis is to think of it. Generally, AK should be considered in all contact lens wearers and in any case of corneal trauma with exposure to soil or contaminated water [20, 23, 36, 54]. Common symptoms are massive pain, photophobia and tearing. The sooner the disease is diagnosed, the better the outcome [6, 12, 54, 99, 104]. If diagnosis is delayed, the amoebae have already penetrated deeply into the corneal stroma and successful therapy becomes exceedingly difficult. AK is usually unilateral and progresses slowly, from epithelial to stromal disease. At the beginning of the infection, a diffuse superficial keratopathy is found, later multifocal infiltrates are almost always observed in the stroma. *Acanthamoeba* sclerokeratitis is an uncommon complication of AK and assumedly has an immune-mediated origin. Tu et al. [104] established five levels of AK severity based on slit-lamp biomicroscopy findings: epitheliitis, epitheliitis with radial neuritis, anterior stromal disease, deep stromal keratitis, or ring infiltrate. The characteristic ring infiltrate is, however, only seen in approximately 50% of patients. In the early stage, AK can easily be confused with *Herpes simplex* keratitis, while in the advanced stage, the infection resembles the clinical picture of a fungal keratitis or a corneal ulcer (Table 1).

**Table 1.** Important characteristics for the differential diagnosis of *Acanthamoeba* keratitis (AK) compared to keratitis due to other infectious agents.

Specific characteristics of AK	When compared to
Pseudo-dendritiform epitheliopathy, epithelium defects without terminal knots, perineural infiltrates, [ring infiltrate]*, endothelium is not involved	*Herpes simplex* keratitis
Usually restricted to cornea, absence of anterior chamber activity, stromal infiltrates are usually multifocal (not monofocal), [ring infiltrate]*	Bacterial keratitis
Usually restricted to cornea, clear epithelium defects, perineural stromal infiltrates, [ring infiltrate]*	Fungal keratitis

* The characteristic ring infiltrate is only seen in the advanced stage and even then only in 50% of patients.

**Contact lens wearers typically seek medical help late, because they are used to minor irritations in the eye.**

The tentative diagnosis of AK can often be made by *in vivo* confocal microscopy (IVCM). The *Acanthamoeba* cysts appearing as hyper-reflective, spherical structures are usually well defined because of their double wall; the trophozoites are difficult to distinguish from leukocytes and keratocyte nuclei [110]. However, the direct detection of the causative agent in a corneal scrape specimen is the only reliable diagnostic method for AK. Culture remains the gold standard of *Acanthamoeba* laboratory diagnosis, but today several PCR-based techniques are also well established and usually increase sensitivity significantly [41, 59, 84, 90]. In cases of severe infection, amoeba density is sometimes very high and the amoebae can already be detected by direct microscopy of the clinical sample, without enrichment. *Acanthamoeba* trophozoites or cysts are readily recognizable in phase contrast microscopy, but also stain well in several stains and cysts exhibit auto-fluorescence [46, 54]. However, particularly if patients have already been pre-treated with antibiotics, amoeba density is usually very low. Moreover, amoebae exhibit altered morphologies – in these cases, even culture often remains negative and molecular techniques are indispensable. Reliable identification below the genus level requires genotyping. Serological techniques are of no diagnostic value as specific antibodies are also detected in apparently healthy people due to the ubiquity of *Acanthamoeba*.

In contrast to infections with other amoebae, acanthamoebae can form cysts within the tissue. As a single cyst surviving in the cornea can lead to reinfection, the progress of therapy should be checked regularly. An ongoing infection should be monitored every 1–2 weeks. After clinical recovery, monthly checks are sufficient, ideally until 6 months after decline of symptoms.

In most countries, the vast majority of AK cases occur in CL wearers and AK can be prevented extensively by strict contact lens hygiene. Typically, singular amoebae gain access to the lens case via tap water or the air, rapidly grow to high densities within the lens case if this is not cleaned properly and regularly, and then attach to the lenses and infect the eye [116]. Wearers of soft contact lenses using multipurpose solutions are at particular risk, because acanthamoebae adhere especially well to the hydrophilic plastic of these lenses, and soft lenses are more difficult to clean than rigid lenses. Moreover, soft lenses are often over worn (dailies used for several days, monthlies used for several months) and are also the type of lenses used by people who do not regularly but only occasionally (e.g. once a week for sports) wear their contacts, and who are often unaware of proper contact lens hygiene. For prophylaxis of AK, lens cases should be cleaned manually and air dried, contact lenses should be cleaned and stored using an appropriate (best: two-step) contact lens cleaning system, and both, lenses and lens cases have to be exchanged regularly.

### 2.1. Material

For confirmation of an AK, sampling and investigation of the correct material is crucial. Only if amoebae are detected in corneal scrapings or in corneal biopsies a reliable diagnosis can be made. Acanthamoebae penetrate the cornea and are usually not found on the corneal surface, thus superficial swab samples or tear samples often remain negative, particularly in the advanced stage of the disease and/or if patients have already been pretreated with antibiotics. On the contrary, contact lens containers, even those of entirely healthy CL wearers, are almost always positive for acanthamoebae, at least in PCR. This means that the detection of *Acanthamoeba* spp. in the CL case does not necessarily indicate an AK. When the CL case is negative, however, it is very unlikely that the patient has an AK, unless, of course, the CL case was recently changed.

The optimal material for AK diagnosis is a corneal scraping/biopsy stored in 200 μL of sterile saline (amoeba saline* or PBS or 0.9% NaCl) in order to prevent desiccation.

*See Table 2.

**Table 2.** Neff’s Amoeba Saline (AS) [71]. 10 mL of each stock solution (10×) are added to 950 mL dH_2_O, mixed, sterilized by filtration and aliquoted into needed volumes.

Stocks (10×)	Grams per 100 mL ddH_2_O
NaCl	1.20
MgSO_4_-7H_2_O	0.04
CaCl_2_ · 2H_2_O	0.04
Na_2_HPO_4_	1.42
KH_2_PO_4_	1.36

### 2.2. Sample preparation

**Figure 2.**
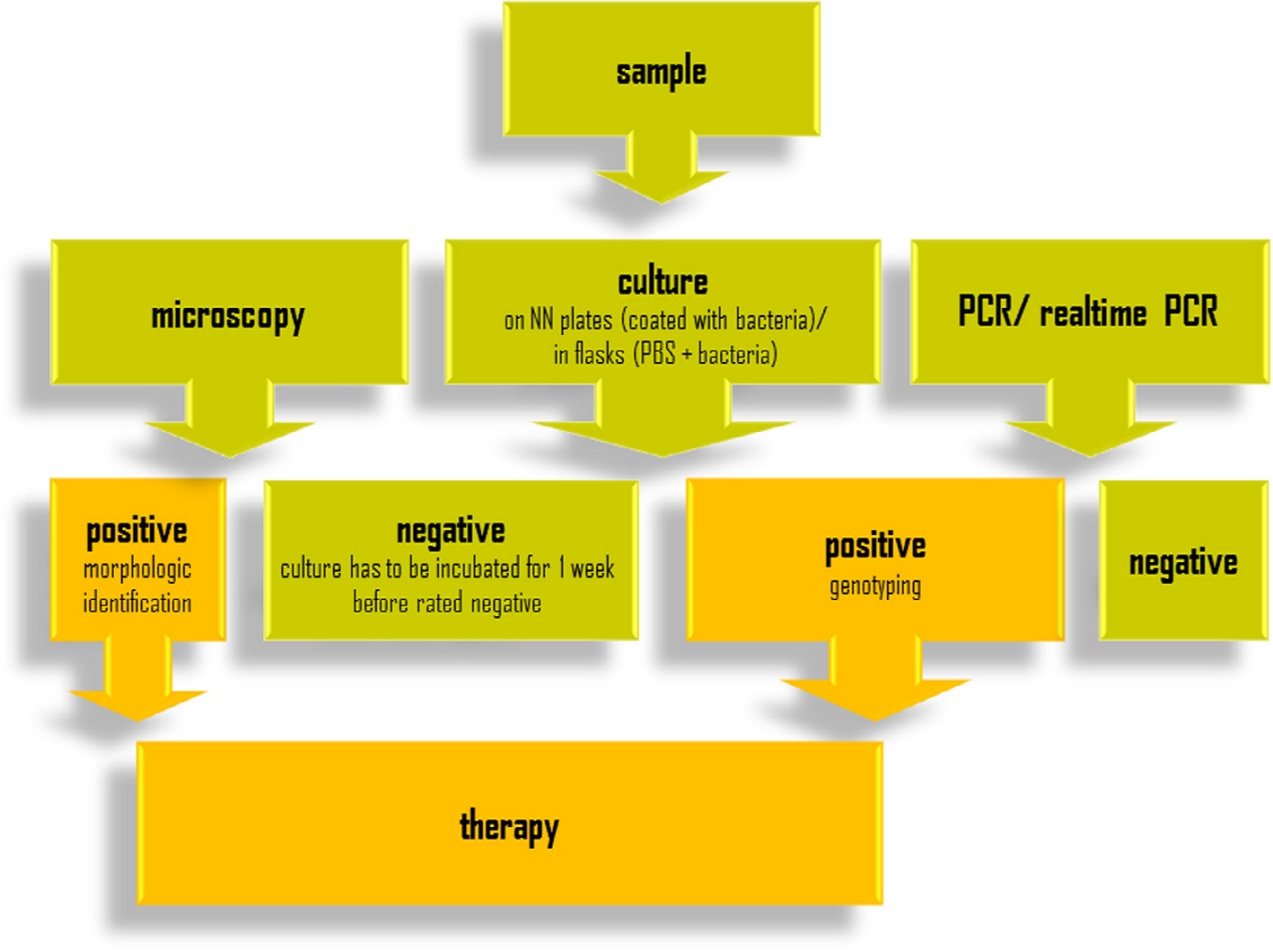
Overview of the diagnostic procedure for *Acanthamoeba* keratitis.

A major challenge in AK diagnostics is the many different types of sample material on the one hand, and sample transport media and containers on the other. Below, we have attempted to provide a guideline for sample preparation depending on the type of material received. A general overview of the diagnostic procedure is given in Figure 2.

When a corneal scraping/biopsy is received, the sample itself is used for DNA isolation, while the transport medium (ideally 200 μL of sterile saline) is used for culture. Larger tissue samples can be cut into two halves, of which one can be transferred onto an agar plate and the other used for DNA isolation. When the sample is received in >200 μL of transport medium, the sample is used for DNA isolation and the transport medium is shaken well, centrifuged at 700 g/7 min, resuspended in 200 μL of sterile saline and processed as described above.

When only liquid is received (e.g. contact lens solution), samples ≤200 μL should be mixed and split into two aliquots directly upon receipt, one aliquot is then used for culture, the other aliquot is used for DNA isolation. Liquid samples >200 μL are centrifuged at 700 g/7 min, resuspended in 200 μL of sterile saline and processed as described above.

Contact lenses or swabs are shaken vigorously in the original transport medium (contact lens solution/sterile saline) and the lens/swab is then inoculated onto an agar plate and the liquid can be used for DNA isolation. When contact lens cases are received, the liquid is processed as described above, but a biofilm swab from the inner surface should also be taken and inoculated onto a plate culture.

When fixed material is received (swabs/contact lens case cell pellets/tissue samples fixed either in ethanol or formalin or embedded in paraffin or as stained sections on microscopic slides), it is recommended to perform staining (lactophenol cotton blue and/or immunostaining) and/or PCR. However, it is important to isolate the DNA using a suitable protocol for the respective material and to adapt the PCR protocol for fragmented DNA, particularly when the material is formalin-fixed (i.e. amplicons should not exceed 300 bp in length).

### 2.3. Direct microscopy

**Figure 3.**
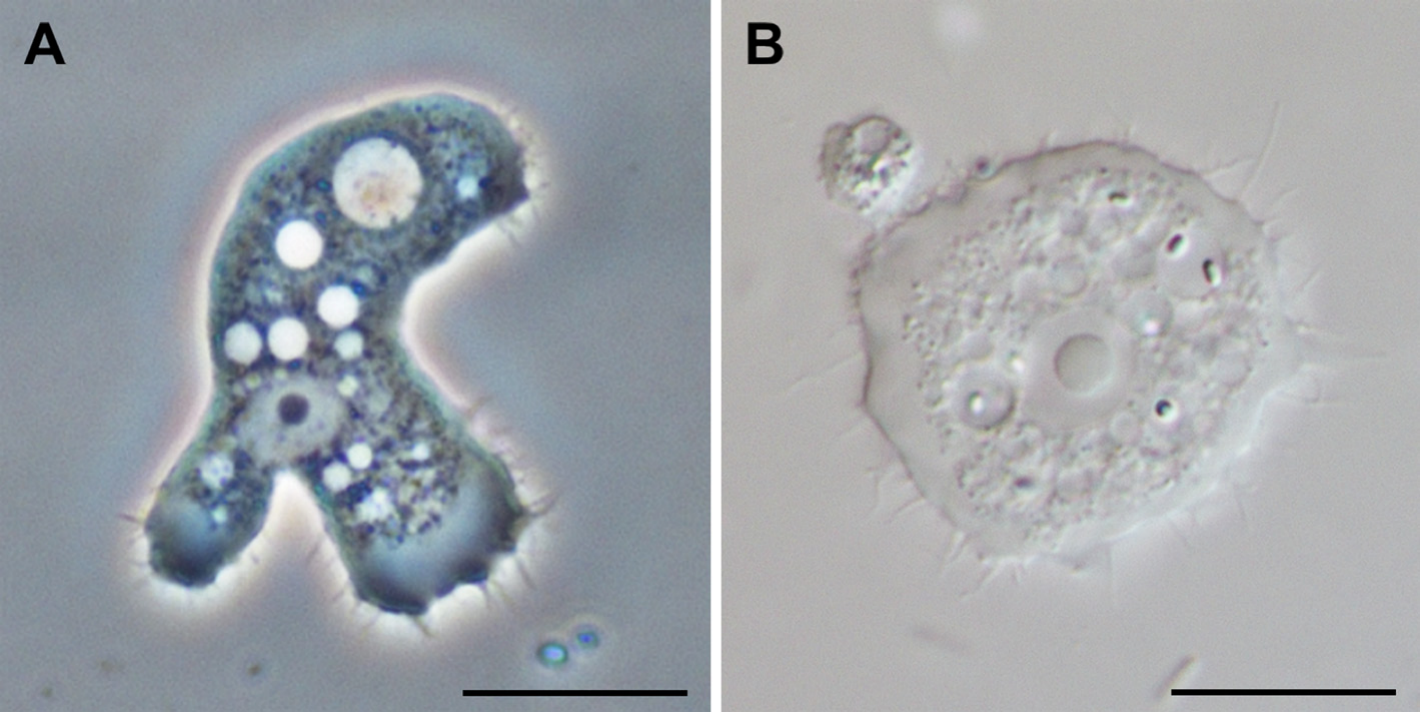
*Acanthamoeba* trophozoites with the characteristic acanthopodia (A) in phase contrast, (B) in bright field microscopy. Scale bar: 10 μm. Originals.

**Figure 4.**
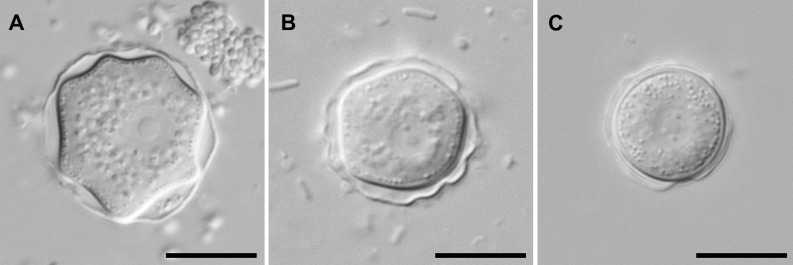
*Acanthamoeba* cysts in interference contrast microscopy (A) morphological group I, (B) morphological group II, (C) morphological group III. Scale bar: 10 μm. Original.

In severe infections or when highly contaminated contact lens cases are investigated, the amoebae can usually already be detected by direct microscopy (200×–400× magnification) of the original sample. For microscopic investigation of amoebae, phase contrast or interference contrast are particularly well suited. Nucleated corneal cells of lower cornea layers may resemble amoebae, but acanthamoebae can be discriminated from other mononuclear cells by their large central nucleolus, their contractile vacuole and their hyaline pseudopodia with characteristic hyaline protrusions, the so-called acanthopodia. The trophozoites are 15–45 μm in size and have an oval to elongated outline (Fig. 3). They move slowly by forming usually one or two pseudopodia in the direction of movement. The cysts are smaller (12–25 μm) and polygonal or star-shaped (Fig. 4). They have two cyst walls which are connected at several points. These points of contact between endocyst and ectocyst are covered by an operculum, which is removed during excystation.

### 2.4. Stains

Stains are practical for the detection of cysts in fresh clinical material or in pelleted lens-case solution and for the investigation of tissue sections. Fast and easy stains are lactophenol-cotton blue or Giemsa, but also calcofluor white and acridine orange usually give very good results. If morphological details are to be studied, it is recommended to use a silver stain, which is particularly well-suited for the investigation of the cysts. However, amoebae have to be cultured prior to staining. A general problem is that other cells, particularly fungi also stain well in these stains. As a specific staining, immunostaining using anti-*Acanthamoeba* antibodies is recommended which is also the stain of choice for tissue sections. Alternatively, tissue section can be stained with haematoxylin & eosin (HE) [35].

#### 2.4.1. Lactophenol-cotton blue (LPCB)

The material is mixed with an adequate volume of LPCB stain (20 g phenol crystals, 20 mL lactic acid, 40 mL glycerol, 0.05 g cotton-blue and 20 mL dH_2_O; or ready-mixed available through e.g. Sigma-Aldrich) and investigated by light microscopy [101]. This stain is particularly well suited for *Acanthamoeba* cysts; the cyst walls and the nucleus appear in an intensive blue, while the cytoplasm stains light-blue.

#### 2.4.2. Acridine orange

Samples are fixed in 95% methanol for 2 min onto a glass slide, air dried, covered with acridine orange staining solution (pH 4) for 2 min, rinsed with H_2_O and air dried. Cysts appear bright orange and are easily discernible in fluorescence microscopy.

#### 2.4.3. Calcofluor white

Samples are transferred to a glass slide, air dried and fixed for 3 min with methanol. Subsequently, the sample is rinsed in PBS and stained using 2–3 drops of calcofluor white solution (0.1%, e.g. Sigma-Aldrich or Thermo Scientific). After 5 min, the slide is rinsed with PBS and counter-stained with Evan’s blue (0.05%, e.g. Sigma-Aldrich or Thermo Scientific) for several seconds. It is important to use an embedding solution without auto-fluorescence. Slides are investigated by fluorescence microscopy (300–440 nm). *Acanthamoeba* cysts appear in a light green because the calcofluor white binds to the cellulose in the cyst walls. Evan’s blue diminishes the background fluorescence making the trophozoites appear reddish-brown.

#### 2.4.4. Silver

The cysts are harvested from a culture plate/flask, suspended in 2 mL amoeba saline (Table 2) and washed three times in amoeba saline by centrifugation (500 g/10 min). The sample is fixed for 20 min in 2% formalin and washed in amoeba saline, the supernatant is removed and the pellet is transferred to a glass slide using an inoculating loop and mixed with Mayer’s albumin (glycerine-albumin 1:1, e.g. Hardy Diagnostics). Then, the cysts are fixed onto the slide using Clarke’s fixative (95% alcohol-acetic acid 9:1) for 2 h. The fixative is removed using dH_2_O and the slides are incubated in 0.5% silver-protein solution in a water bath at 60 °C. After 2 h, the slides are transferred to the reducing agent (1% hydroquinone in 5% Na_2_SO_3_) and incubated during gentle shaking for several seconds up to 5 min. The slides are washed in dH_2_O, dehydrated in an alcohol series, cleared with xylene, mounted and investigated by bright field microscopy.

#### 2.4.5. Immunostain

To the best of our knowledge, no commercial kit is available, but antisera against the three *Acanthamoeba* groups (I–III), produced by immunization of a rabbit with *Acanthamoeba* whole-cell antigen, are available in many laboratories (including our own) and can be obtained upon request.

#### 2.4.6. Haematoxylin & eosin (HE)

The tissue section is fixed in 10% neutral buffered formalin solution (e.g. Sigma-Aldrich). Serial sections of 6 μm are produced, deparaffinized for 1–2 min in xylene, dehydrated in alcohol and washed with dH_2_O. Subsequently, the sample is stained with haematoxylin and eosin, washed, covered with a cover slip and investigated by bright field microscopy.

### 2.5. Culture

**Figure 5.**
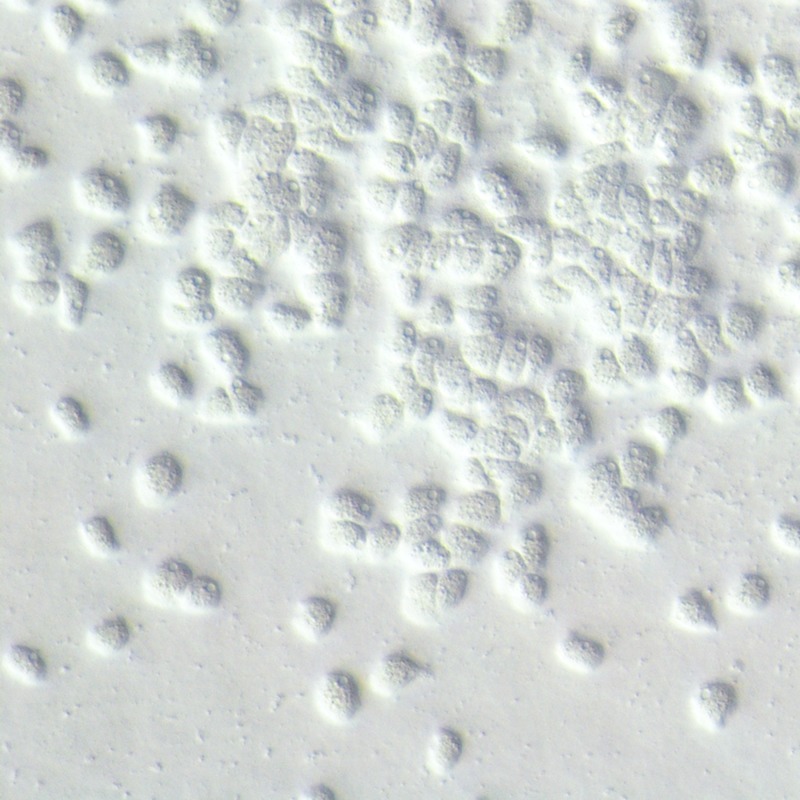
*Acanthamoeba* trophozoites observed in culture in a case of severe AK infection.

The gold standard for *Acanthamoeba* detection is still the plate culture technique [71, 91]. The material (corneal scrapings/biopsies or transport medium/contact lenses/swabs, etc.) is applied centrally onto a 90 mm 1.5% non-nutrient (NN) agar plate covered with a lawn (100 μL) of a 24 h old culture of non-mucous bacteria (e.g. *Escherichia coli*). Plates are sealed with Parafilm^®^, incubated at 30 °C and screened daily for amoebae, optimally by inverted phase contrast microscopy. In cases of severe infection, amoebae are usually already visible after 24–48 h (Fig. 5). However, samples should be observed for up to 1 week to reliably prove a negative result. Alternatively, amoebae can be cultured in tissue culture flasks in a suspension of bacteria in PBS.

In culture, acanthamoebae form cysts within approximately 1 week (depending on temperature and availability of nutrients). These cysts can be identified at least down to the morphological group level (*Acanthamoeba* sp. group I–III) based on size, morphology and number of opercula [80] (Fig. 4). Most AK cases are caused by representatives of group II (Fig. 4B), but group III strains have also been described as causative agents of AK. Group II strains have polygonal cysts with 3–7 cyst arms, while group III strains are rounded and do not have clearly visible cyst arms. Group I strains with their large and beautifully star-shaped cysts have not (yet?) been described to cause AK. Species identification can be achieved using the identification key by Page [71]. However, in some cases, morphological identification is ambiguous and the validity of some described species has been questioned altogether.

If the isolated amoebae are needed for further studies (e.g. genotyping), sub-cultures should be prepared since fungi and other microorganisms also grow very well in these cultures. In addition, several physiological properties can be used to further describe and discriminate *Acanthamoeba* isolates, including growth rate, temperature tolerance, cell culture pathogenicity and *in vivo* mouse pathogenicity [22, 24, 34, 115].

#### 2.5.1. Sub-culture

From positive samples, clonal cultures can be prepared by transferring a small piece (<1 cm^2^) of agar with only few clean cysts on it (optimally a single cyst using a micromanipulator) upside down to a fresh plate. Plates should be sub-cultured every 2–4 weeks.

Monoxenic plates sealed with Parafilm^®^ can be kept for several months at room temperature. If they do not entirely dehydrate, cysts remain viable for many years.

##### 2.5.1.2. Temperature tolerance

Sub-cultures can be used for investigating the temperature tolerance of the respective isolate. Parallel cultures are incubated at 30 °C, 37 °C and 42 °C, respectively. The temperature of the human eye is approximately 34 °C. Usually however, the ability to grow at 37 °C (body temperature) and 42 °C (high fever) is also investigated. Plates are investigated daily by phase contrast microscopy.

#### 2.5.2. Axenisation

Acanthamoebae can be axenised by harvesting cysts from the plate cultures and incubating them in 3% HCl overnight in order to eliminate the bacteria. It is usually sufficient to install three parallel plate cultures and wait for cyst formation (usually approximately 2 weeks). It is important that cysts be fully mature, because otherwise they will not survive the acid treatment. Subsequently, the cysts are washed 2–3× in amoeba saline (700 g/7 min) to remove remaining acid and transferred to liquid cultures. An easy culture medium for acanthamoebae is proteose peptone-yeast extract-glucose (PYG) [71] (Table 3).

**Table 3.** PYG medium [71]. Compounds are weighted into a 1 L bottle, filled up to 1 L with dH_2_O, mixed and sterilized by filtration.

Compound	Grams
Proteose-Peptone	10.00
Glucose	18.00
NaCl	1.20
MgSO_4_-7H_2_O	0.04
CaCl_2_ · 2H_2_O	0.04
Na_2_HPO_4_	1.42
KH_2_PO_4_	1.36

To keep axenic cultures running, medium has to be changed ideally every 1–2 weeks. The cultures should be checked regularly for bacterial contamination (e.g. by transferring an aliquot of the supernatant to bacterial broth), as should the amoebae themselves for endocytobionts. To reduce the risk of contamination, antibiotics (e.g. 200 IU penicillin and 200 μg/mL streptomycin) and/or antimycotics (e.g. amphotericin B) can be added to the culture medium.

Liquid culture is not suited to initial clinical samples, as bacteria and fungi would overgrow the cultures (a clinical sample from the eye surface is never sterile).

#### 2.5.3. Cell culture pathogenicity

Trophozoites are harvested from axenic cultures by centrifugation (700 g/7 min.) and transferred onto a monolayer of human (e.g. HeLA, HEp-2 or keratinocytes) or animal cells (e.g. VERO) in an amoeba/cell ratio of 1:10. The amoebae are designated as highly cytopathic, when the monolayer is completely lysed within 24 h.

#### 2.5.4. Mouse inoculation

Trophozoites are harvested from axenic cultures by centrifugation (700 g/7 min.), re-suspended in sterile PBS and inoculated into mice intra-nasally or intra-cerebrally. Young mice are generally more susceptible to an *Acanthamoeba* infection. Pathogenic strains lead to death within a few days up to 4 weeks. Importantly, amoebae can lose their pathogenicity during long-term axenic laboratory culture.

### 2.6. DNA isolation

For genotyping, actively growing amoebae (~10^6^ cells) are harvested from culture plates and resuspended in 100 μL of sterile 0.9% NaCl for DNA isolation. Whole-cell DNA can be isolated from the amoebal suspensions using a commercial DNA isolation kit following the manufacturer’s protocol for the respective type of material. When larger tissue samples are received, we recommend homogenization of the material prior to DNA isolation.

### 2.7. PCR/real-time PCR

The most frequently used PCR for *Acanthamoeba* diagnostics is probably the one established by Schroeder et al. [90, 97] amplifying a fragment of the 18S rRNA gene using the JDP1 (5′-GGCCCAGATCGTTTACCGTGAA-3′) and JDP2 (5′-TCTCACAAGCTGCTAGGGAGTCA-3′) primers. In this PCR, the length of the amplicon varies between 423 and 551 bp depending on the genotype, and DNA sequencing of the amplicon allows for genotyping in most cases. Generally, whichever diagnostic PCR is used, it should be run with at least two different dilutions from each sample (as the proportion amoebal DNA: human DNA can vary greatly) and a genotype T4 reference strain should be used as a positive and DNA-free water as a negative control. Amplicons are visualized by agarose-gel electrophoresis and, if genotyping is required, the respective bands are extracted from the gel, purified and subjected to DNA sequencing.

For samples that had been fixed in formaldehyde, we employ a modified PCR using the JDP1 primer from the PCR described above and the P2r primer (5′-GACTACGACGGTATCTGATC-3′) [113], which amplifies a shorter (~300 bp) fragment of the 18S rRNA gene.

In the past years, several protocols for real-time PCR have also been published [43, 61, 81, 84]. A highly sensitive and specific assay is the multiplex real-time PCR established by Qvarnstrom et al. [81], which for AK diagnostics can also be run as a singleplex.

### 2.8. Genotyping

Sequences of the PCR amplicons can be obtained by direct sequencing or by cloning. Generally, it is recommended to obtain sequences from both strands and assemble them to give a consensus sequence. For genotyping, obtained sequences are compared to sequences of *Acanthamoeba* reference strains by multiple alignments with all available genotypes at that time (currently 19) with the model assumption of a <5% sequence dissimilarity within one genotype as established by Gast et al. [33] and Stothard et al. [97]. Worldwide, the vast majority of AK cases are caused by *Acanthamoeba* genotype T4, but genotypes T3 and T11 are also commonly associated with AK, and in fact most genotypes known to date have at least once been involved in an AK case [11, 116].

## 3. Pathogenesis of *Acanthamoeba* keratitis

**Figure 6.**
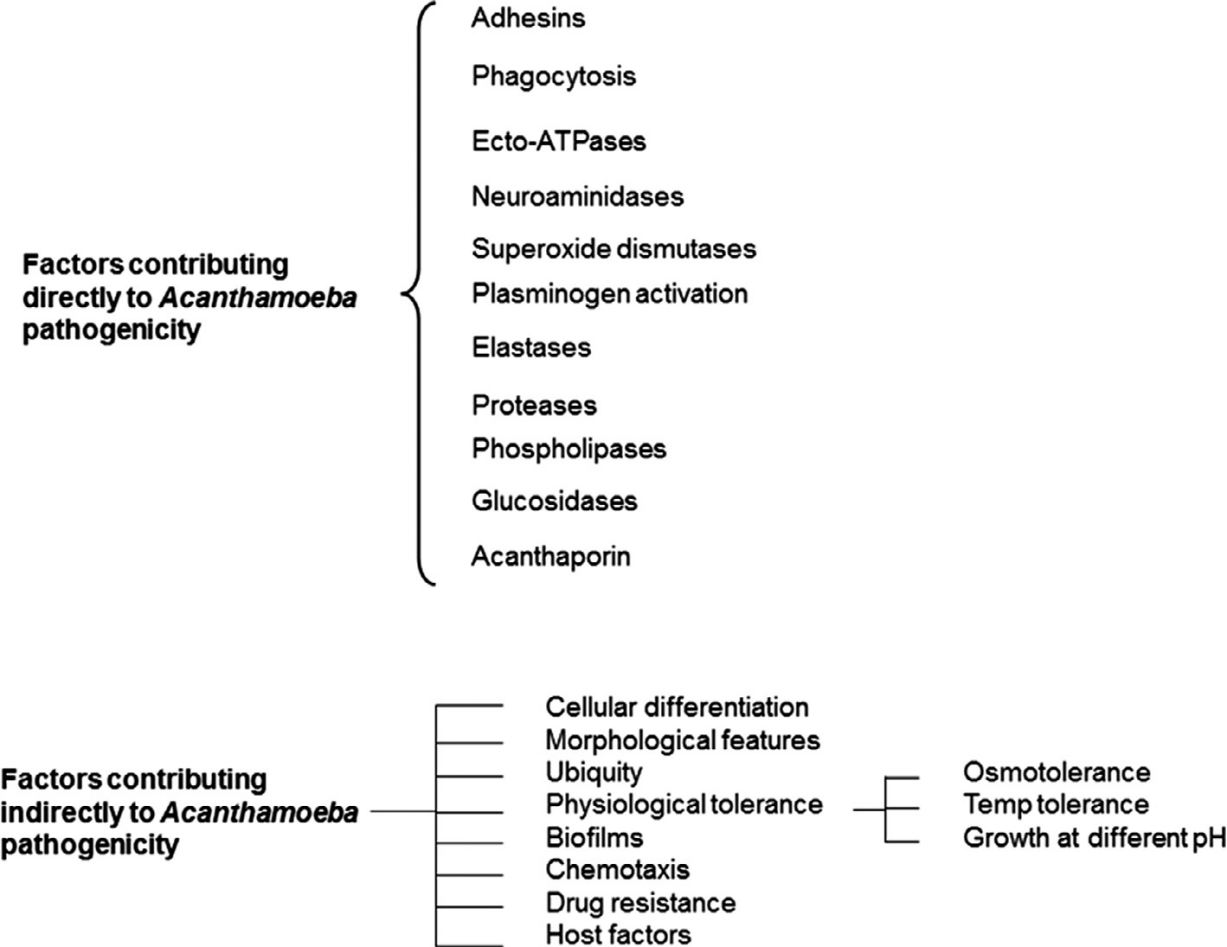
Factors contributing to the pathogenicity of *Acanthamoeba*.

The devastating nature of *Acanthamoeba* keratitis and the problems associated with its diagnosis and successful therapy suggest a need for complete understanding of the pathogenesis and pathophysiology to find alternative therapeutic interventions. Another major concern during the course of therapy is the ability of *Acanthamoeba* to transform into dormant cyst forms, which may resist recommended levels of antimicrobial chemotherapy. The ability of *Acanthamoeba* to produce infection requires specific adhesins, production of toxins, and its ability to resist immune/environmental factors and chemotherapeutic agents, which likely enable this pathogen to produce infection. For simplicity, the information is divided into factors contributing directly and indirectly to *Acanthamoeba* pathogenicity (Fig. 6).

### 3.1. Factors contributing directly to the pathogenicity of *Acanthamoeba*

#### 3.1.1. Adhesion

**Figure 7.**
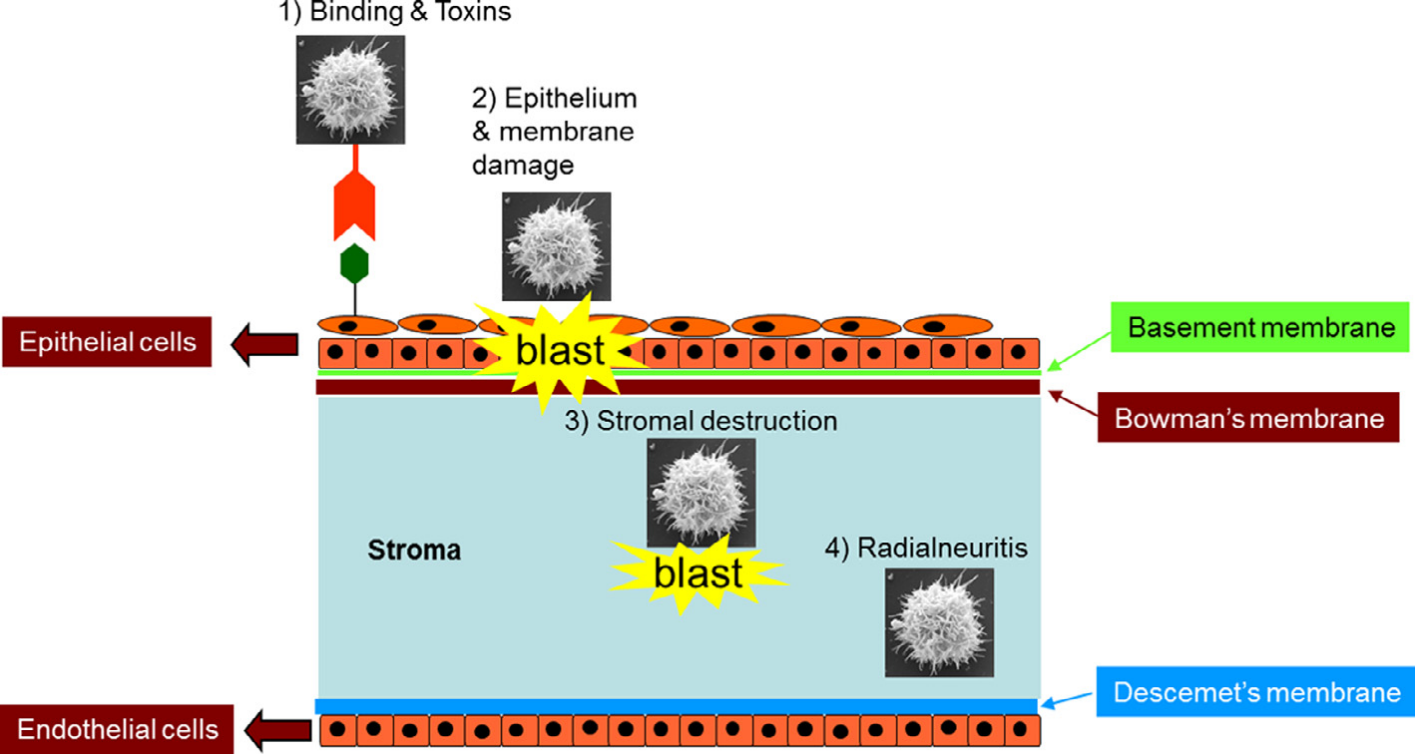
Schematics of *Acanthamoeba*-mediated corneal damage. “Blast” refers to damage.

Adhesion is an important step in the pathogenic cascades of *Acanthamoeba* keratitis leading to secondary events and amoebae crossing biological barriers (Fig. 7). Several adhesins have been identified in *Acanthamoeba*, including a mannose-binding protein [30], a laminin-binding protein with a predicted molecular mass of 28.2 kDa [40] and a 55 kDa laminin-binding protein [87]. Notably, oral immunization with recombinant mannose-binding protein ameliorates *Acanthamoeba* keratitis in the Chinese hamster model [30, 31], and has shown that the mannose-binding protein gene in *Acanthamoeba* contains six exons and five introns that span 3.6 kbp. The 2.5 kbp cDNA codes for an 833 amino acids precursor protein with a signal sequence (residues 1–21 aa), an N-terminal extracellular domain (residues 22–733 aa) with five N- and three O-glycosylation sites, a transmembrane domain (residues 734–755 aa), and a C-terminal intracellular domain (residues 756–833 aa).

**Figure 8.**
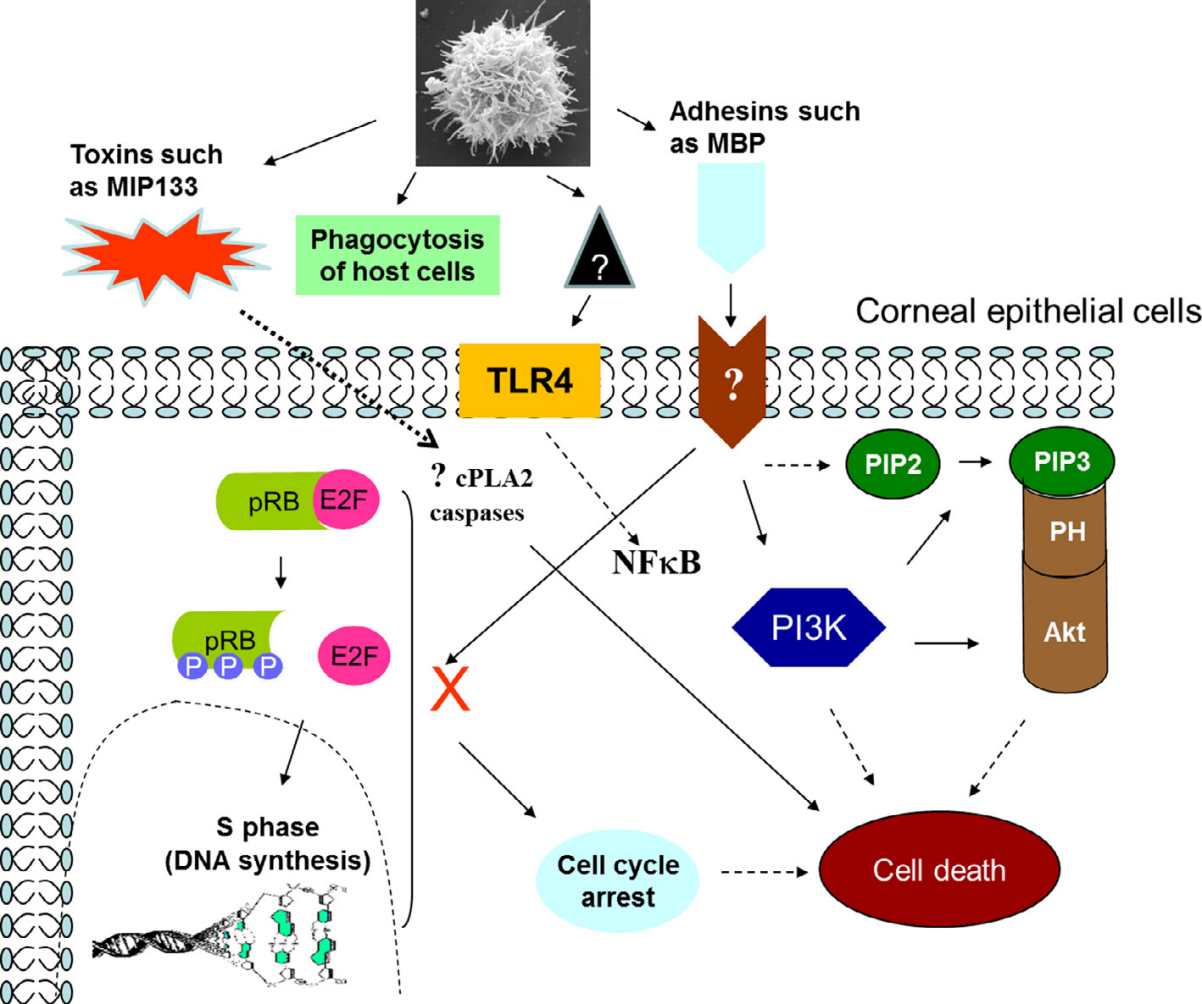
*Acanthamoeba*-mediated corneal epithelial cell death.

On the host side, parasite binding to specific host cell receptor(s) remains incompletely understood. However Toll-like receptor-4 (TLR-4) is shown to provide a docking site for *Acanthamoeba* [82, 83]. Complete identification of adhesins involved in binding to various cell types, tissues and surfaces together with specific receptor(s) is a largely unexplored area, offering tremendous research opportunities. The binding of *Acanthamoeba* to host cells interferes with the host intracellular signalling pathways. For example, TLR activation leads to TLR4-Myeloid differentiation primary response gene 88 (MyD88)-Nuclear Factor-Kappa B (NF-kappaB) and TLR4-Extracellular signal-regulated kinases1/2 (ERK1/2) pathways [82, 83]. This was confirmed using anti-TLR antibodies or specific inhibitors pyrrolidinedithiocarbamate (PDTC) (for the NF-kappa B pathway) and U0126 (for the ERK pathway). Using cell cycle microarrays, it has been shown that adhesion of *Acanthamoeba* to host cells regulates the expression of a number of genes important for the cell cycle such as GADD45A and p130 Rb, associated with cell cycle arrest, as well as inhibiting the expression of other genes, such as those for cyclins F, G1 and cyclin dependent kinase-6 that encode proteins important for cell cycle progression [95]. *Acanthamoeba* inhibited pRb phosphorylation (a master regulator of cell cycle) in human corneal epithelial cells, indicating that *Acanthamoeba* induces cell cycle arrest in the host cells. *Acanthamoeba*-mediated host cell death is dependent on the activation of phosphatidylinositol 3-kinase [96]. This was shown using LY294002, a specific phosphatidylinositol 3-kinase inhibitor, which blocked *Acanthamoeba*-mediated host cell death. These findings were further confirmed using host cells expressing dominant negative p85, i.e. a regulatory subunit of phosphatidylinositol 3-kinase. The host cells expressing dominant negative phosphatidylinositol 3-kinase were significantly less susceptible to *Acanthamoeba*-mediated damage (Fig. 8). Chusattayanond et al. [16] have shown that host cell apoptosis induced by *Acanthamoeba* is caspase-dependent, mediated by over-expression of pro-apoptotic proteins in the mitochondrial pathway, while Tripathi et al. [102] demonstrated the role of the cytosolic phospholipase A2α (cPLA2α) pathway in host cell apoptosis.

#### 3.1.2. Phagocytosis

Adhesion of *Acanthamoeba* leads to secondary processes such as phagocytosis or secretion of toxins. The primary role of *Acanthamoeba* phagocytosis is to take up food particles. However, the ability of *Acanthamoeba* to form food cups or amoebastomes during incubations with the host cells suggests it has a role in the pathogenesis of *Acanthamoeba* [26, 47, 76]. Within 40 s, bound particles are surrounded by pseudopods, brought into the cytoplasm, and released as phagosome into the cytoplasmic stream. The oxidative metabolism in *Acanthamoeba* has some remarkable similarities to the respiratory burst oxidase of neutrophils [13]. Cytochalasin D, an inhibitor of actin polymerization blocked *Acanthamoeba*-mediated host cell death, confirming that actin-mediated cytoskeletal rearrangements play an important role in the pathogenesis of *Acanthamoeba* [70, 100]. Genistein (a protein tyrosine kinase inhibitor) inhibited, while sodium orthovanadate (protein tyrosine phosphatase inhibitor), stimulated *Acanthamoeba* phagocytosis, indicating that tyrosine kinase-induced actin polymerization is important in *Acanthamoeba* phagocytosis [3]. Rho kinase inhibitor, Y27632, partially blocked *Acanthamoeba* phagocytosis. Y27632 is known to block stress fibre formation by inhibiting myosin light chain phosphorylation and cofilin phosphorylations but independent of the profilin pathway. LY294002, a specific inhibitor of phosphatidylinositol 3-kinase, inhibited *Acanthamoeba* phagocytosis. Inhibition of Src kinase using a specific inhibitor, PP2 (4-amino-5-(4 chlorophenyl)-7-(t-butyl)pyrazolo [3,4-d] pyrimidine) but not its inactive analog, PP3 (4-amino-7-phenylpyrazolo[3,4-d] pyrimidine), hampered the phagocytic ability of *A. castellanii* [93]. The precise elucidation of molecular mechanisms associated with *Acanthamoeba* phagocytic pathways will be of value in the development of therapeutic interventions.

#### 3.1.3. Ecto-ATPases

Ecto-ATPases are glycoproteins expressed in the plasma membranes with their active sites facing the external medium. Ecto-ATPases hydrolyze extracellular ATP and other nucleoside triphosphates. The resultant ADP can have toxic effects on the host cells. For example, it has been shown that ADP released by *Acanthamoeba* bind to P2y2 purinergic receptors on the host cells, causing an increase in intracellular calcium, inducing caspase-3 activation and finally resulting in apoptosis [59]. A P2 receptor antagonist, suramin, inhibited *Acanthamoeba*-mediated host cell death [59], suggesting that ecto-ATPases play an important role in *Acanthamoeba* pathogenesis in a contact-independent mechanism. The clinical isolates of *Acanthamoeba* exhibited higher ecto-ATPase activities compared with weak pathogenic isolates [94]. Several ecto-ATPases of approximate molecular weights of 62, 100, 218, 272 and more than 300 kDa have been described in *Acanthamoeba*. However, further research is needed to elucidate their function in *Acanthamoeba* biology, and investigate their precise role in *Acanthamoeba* pathogenesis.

#### 3.1.4. Neuraminidase activity

*Acanthamoeba* exhibited neuraminidase activity. The enzyme activity is optimal at pH 5 and at temperatures of 25–30 °C. The live amoebae released sialic acid from the human cells. Therefore, the neuraminidase of *Acanthamoeba* could be relevant in the colonization of amoebae, and important in producing damage of the sialic acid-rich corneal epithelium. Interestingly, neuraminidases of *Trypanosoma cruzi* and *Acanthamoeba* are immunologically related, as demonstrated by antibodies against neuraminidase of *Trypanosoma cruzi*, which reacted with *Acanthamoeba* in immunofluorescence, immunoblotting and enzyme-linked immunosorbent assays [73, 74].

#### 3.1.5. Superoxide dismutase

The enzyme superoxide dismutase catalyzes the dismutation of superoxide into oxygen and hydrogen peroxide. It is an important antioxidant defence exposed to oxygen. Superoxide is one of the main reactive oxygen species in the cell and as such, superoxide dismutase plays an important antioxidant role. Two superoxide dismutases have been identified in *Acanthamoeba*: an iron superoxide dismutase (approximate molecular weight of 50 kDa) and a copper-zinc superoxide dismutase (approximate molecular weight of 38 kDa). These enzymes occur as cytoplasmic and detergent-extractable fractions. They may be potential virulence factors of *Acanthamoeba* by acting both as anti-oxidants and anti-inflammatory agents. They may also provide additional targets for chemotherapy and immuno-diagnosis of *Acanthamoeba* infections [15]. *A. castellanii* iron superoxide dismutase may play essential roles in the survival of amoebae not only by protecting themselves from endogenous oxidative stress, but also by detoxifying oxidative killing of amoebae by host immune effector cells [50].

#### 3.1.6. *Acanthamoeba*-induced plasminogen activation

*Acanthamoeba* displayed plasminogen activator activity by catalyzing the cleavage of host plasminogen to form plasmin, which can activate host proteolytic enzymes, such as pro-matrix metalloproteases. Once activated, the matrix metalloproteases degrade the basement membranes and the components of the extracellular matrix such as type I and type II collagens, fibronectins and laminin. Thus, the matrix metalloproteinases are involved in tissue remodelling. The pathogenic *Acanthamoeba* showed positive chemotactic response to the endothelial extracts [109].

#### 3.1.7. Elastase

*Acanthamoeba* is known to produce elastase with broad specificity. Moreover, elastases are known to degrade a range of connective tissue proteins such as elastin, an elastic fibre, fibrinogen, collagen, and proteoglycans, which together determined the mechanical properties of the connective tissue. Tissues altered by prior elastase treatment are more susceptible to oxygen radical attack, suggesting their involvement in the pathogenesis and pathophysiology of *Acanthamoeba* infections [18, 46, 47, 54]. The elastases were in the region of 70–130 kDa and serine peptidases were found to be possible elastase candidates [28].

#### 3.1.8. Proteases

Proteases are degradative enzymes that catalyze the total hydrolysis of proteins. *Acanthamoeba* is shown to exhibits proteolytic activities. The primary role of *Acanthamoeba* proteases is to degrade food substances for feeding purposes. Pathogenic *Acanthamoeba* exhibit increased extracellular protease activities. The link between pathogenicity and the increased levels of extracellular proteases suggests that pathogenic *Acanthamoeba* utilize proteases to facilitate invasion of the host. *Acanthamoeba* is known to produce serine, cysteine and metalloproteases. Several serine proteases have been identified ranging in molecular weights from >20 kDa to >200 kDa. They are shown to possess collagen degradation activity, plasminogen activator, and degradation of fibronectin, fibrinogen, IgG, IgA, albumin, haemoglobin, protease inhibitors, interleukin-1, chemokines and cytokines [46, 54, 55, 60]. A 133 kDa serine protease, called MIP133 has been identified as a crucial component of the pathogenic cascade of *Acanthamoeba* pathogenesis. The MIP133 serine protease is shown to induce the degradation of keratocytes, iris ciliary body cells, retinal pigment epithelial cells, corneal epithelial cells and corneal endothelial cells, and induce apoptosis in macrophage-like cells. The properties of serine proteases facilitate *Acanthamoeba* invasion of the corneal stroma, leading to secondary reactions such as oedema, necrosis and inflammatory responses. A direct functional role of serine proteases in *Acanthamoeba* infections is indicated by the observations that intrastromal injections of *Acanthamoeba* conditioned medium produced corneal lesions *in vivo*, similar to those observed in *Acanthamoeba* keratitis patients, and this effect is inhibited by phenylmethylsulfonyl fluoride, a serine protease inhibitor [37, 67, 68]. In addition, the chemically synthesized siRNA against the catalytic domain of the extracellular serine proteases of *Acanthamoeba* reduced protease activity and *Acanthamoeba*-mediated host cell cytotoxicity. These results support the idea that extracellular serine proteases are directly involved in the pathogenesis and virulence of *Acanthamoeba* [55]. In addition, several cysteine proteases have been identified in *Acanthamoeba*, including 43, 65, 70 and 130 kDa cysteine proteases [46, 47]. In addition to serine and cysteine proteases, there is evidence for metalloprotease activity in *Acanthamoeba*. An 80 kDa metalloprotease was identified in co-cultures of *Acanthamoeba* and host cells, but its origin (whether *Acanthamoeba* or the host cells) was not established. Later studies identified a 150 kDa extracellular metalloprotease from *Acanthamoeba* isolate of the T1 genotype. This metalloprotease exhibited properties of extracellular matrix degradation, as demonstrated by its activity against collagen I and III (major components of the collagenous extracellular matrix), elastin (elastic fibrils of the extracellular matrix), plasminogen (involved in proteolytic degradation of the extracellular matrix), as well as degradation of casein, gelatine and haemoglobin.

Recently, the complete sequence of a type-1 metacaspase from *Acanthamoeba* was reported, comprising 478 amino acids [102]. Later studies revealed that *A. castellanii* metacaspases associate with the contractile vacuole and have an essential role in cell osmoregulation suggesting its attractiveness as a possible target for treatment therapies against *A. castellanii* infection [89]. These studies showed that *Acanthamoeba* exhibited diverse proteases and elastases, which could play important roles in *Acanthamoeba* infections. The precise mechanisms of protease mode of action at the molecular level are only beginning to emerge. Proteases have been shown to be “druggable” targets, as evidenced by the widespread use of protease inhibitors as effective therapy for hypertension and AIDS, and the current clinical development of protease inhibitors for diabetes, cancer, thrombosis, and osteoporosis. As long as issues such as the difficulty of achieving selectivity can be addressed through targeting allosteric sites, protease-based drug therapy has tremendous potential in the treatment of many infectious diseases. Future studies will further determine the role of proteases as vaccine targets, search for novel inhibitors by screening of chemical libraries, or rational development of drugs based on structural studies should enhance our ability to target these important pathogens.

#### 3.1.9. Phospholipases

During phagocytosis, there is a large turnover of the plasma membrane in *Acanthamoeba*, indicating that there is controlled local degradation of phospholipids leading to instability of the membrane phospholipid bilayer, which would then reform after the acylation of the lysophospholipid. In support of this, all of the enzymes that are needed for such a cycle are present in the plasma membrane of *Acanthamoeba*, including phospholipase A2, acyl CoA:lysolecithin acyltransferase, and acyl CoA synthetase. Phospholipase A1 and lysophospholipase are also present in the plasma membrane of *Acanthamoeba*. The plasma membrane lysophospholipase may also serve to protect the cell from the lytic effect of lysophospholipids either of exogenous or endogenous origin. The plasma membranes have the enzymatic capability of modulating the fatty acyl composition of phospholipids by de-acylation and acylation. Our knowledge of phospholipases in the virulence of *Acanthamoeba* is fragmented, however several studies have shown that pathogenic *Acanthamoeba* that exhibit cytopathic effects on mammalian cells *in vitro* liberate more phospholipase, suggesting their possible involvement in *Acanthamoeba* infections. Because phospholipases cleave phospholipids, it is reasonable to suspect that they play a role in membrane disruptions, penetration of host cells, and cell lysis. However this remains to be determined. Other actions of phospholipases may involve interference with intracellular signalling pathways. Phospholipases generate lipids and lipid-derived products that act as second messengers. *A. castellanii* lysates and their conditioned medium exhibited phospholipase activities [66]. Sphinganine, a PLA2 inhibitor showed robust amoebistatic properties but had no effect on the viability of *A. castellanii*. These studies suggest that *Acanthamoeba* phospholipases and/or lysophospholipases may play a role in producing host cell damage or affect other cellular functions such as induction of inflammatory responses, thus facilitating *Acanthamoeba* virulence. More studies are needed to identify and characterize *Acanthamoeba* phospholipases and to determine their potential role in the development of therapeutic intervention. This is not a novel concept: earlier studies have shown that phospholipase C from *Clostridium perfringens* induced protection against *C. perfringens*-mediated gas gangrene. In addition, targeting of phospholipases using synthetic inhibitor compounds has been shown to prevent *Candida* infections. Antibodies produced against *Acanthamoeba* phospholipases may also be of potential value in the development of sensitive and specific diagnostic assays as well as of therapeutic value [42].

#### 3.1.10. Glycosidases (also called glycoside hydrolases)

Glycoside hydrolases catalyze the hydrolysis of glycosidic linkage to generate smaller sugars. Glycoside hydrolases are ubiquitous in nature and involved in the degradation of biomass such as cellulose and in a variety of cellular functions. Together with glycosyltransferases, glycosidases form the major catalytic machinery for the synthesis and breakage of glycosidic bonds. *Acanthamoeba* exhibits glycosidase activities including beta-glycosidase, alpha-glucosidase, beta-galactosidase, beta-N-acetyl-glucosaminidase, beta-N-acetyl-galactosaminidase and alpha-mannosidase [38, 39]. *Acanthamoeba* extracts mediate enzymatic lysis of cell walls from several species of bacteria including *Micrococcus lysodeikticus*, *Micrococcus roseus*, *Streptococcus faecalis*, *Bacillus megaterium*, *Sarcina lutea*, *Micrococcus radiodurans* and limited activity against *Bacillus subtilis*, *Bacillus cereus*, but has no effects on *Acanthamoeba* cyst walls or chitin. Exhaustive digestion of *Micrococcus lysodeikticus* cell walls released free N-acetyl-glucosamine, N-acetyl-muramic acid, glycine, alanine, glutamic acid and lysine, suggesting that *Acanthamoeba* possesses both endo- and exo-hexosaminidases and beta-N-acetyl-hexosaminidases. Since *Acanthamoeba* is known to utilize maltose, cellobiose, sucrose or lactose, some of the glycosidases indicated above may suggest the utilization of these disaccharides [42].

#### 3.1.11. Acanthaporin

Recently, acanthaporin, the first pore-forming toxin was described from *Acanthamoeba* [64]. Acanthaporin was isolated from extracts of virulent *A. culbertsoni* by tracking its pore-forming activity, molecularly cloning the gene of its precursor, and recombinant expression of the mature protein in bacteria. Acanthaporin was cytotoxic for human neuronal cells and exerted antimicrobial activity against a variety of bacterial strains by permeabilizing their membranes [64]. The tertiary structures of acanthaporin’s active monomeric form and inactive dimeric form, both solved by NMR spectroscopy, revealed a currently unknown protein fold and a pH-dependent trigger mechanism of activation.

### 3.2. Factors contributing indirectly to *Acanthamoeba* pathogenicity

The ability of *Acanthamoeba* to produce human diseases is a multifactorial process and is, amongst other factors, dependent on its ability to survive outside its host and under diverse conditions (high osmolarity, varying temperatures, food deprivation and resistance to chemotherapeutic drugs) [24, 48, 106, 107, 114]. The ability of *Acanthamoeba* to overcome such conditions can be considered as contributory factors towards disease and are indicated below.

#### 3.2.1. Morphological features

The infective forms of *Acanthamoeba* or trophozoites do not have a distinct morphology. However, they do possess spine-like structures known as acanthopodia on their surface, which allow them to modulate binding of *Acanthamoeba* to biological and inert surfaces. In addition, their amoeboid motion resembles that of macrophages/neutrophils and it is likely that *Acanthamoeba* employ similar strategies to traverse biological barriers and invade tissues using the paracellular route.

#### 3.2.2. Temperature tolerance, osmotolerance and growth at different pH

Being a free-living amoeba, *Acanthamoeba* is exposed to various temperatures, osmolarity and pH. Similarly contact with tear film exposes *Acanthamoeba* to high osmolarity (due to salinity in tears), high temperatures as well as alterations in pH. For successful transmission, *Acanthamoeba* must withstand such stress and exhibit biological activity. Pathogenic *Acanthamoeba* showed high levels of heat shock proteins (i.e. HSP60 and HSP70) compared with weak pathogens [75, 77]. The higher levels of heat shock proteins in *Acanthamoeba* may indicate their involvement in (i) tolerance to hosts’ stressors and/or (ii) in species’ virulence [75]. The ability of *Acanthamoeba* to grow at high temperatures and high osmolarity correlates with the pathogenicity of *Acanthamoeba* isolates, and may provide a good indicator of the pathogenic potential of a given isolate [46, 47, 54]. The precise mechanisms by which pathogenic *Acanthamoeba* adapt to higher temperatures and maintain their metabolic activities require further studies.

#### 3.2.3. Cellular differentiation

Cellular differentiation is the ability of *Acanthamoeba* to differentiate into a morphologically distinct dormant cyst form or a vegetative trophozoite form. This is a reversible change, dependent on environmental conditions. Cysts are resistant to various antimicrobial agents and adverse conditions such as extremes in temperatures, pH, osmolarity, desiccation and cysts can be airborne: all of which presents a major problem in chemotherapy because their persistence may lead to recurrence of the disease. Furthermore, *Acanthamoeba* cysts can survive several years while maintaining their pathogenicity [62]. These characteristics suggested that the primary functions of cysts lie in withstanding adverse conditions and in the spread of amoebae throughout the environment. In addition, this may represent the ability of *Acanthamoeba* to alternate expression of surface proteins/glycoproteins, in response to changing environments and/or immune surveillance. Cellular differentiation represents a major factor in the transmission of *Acanthamoeba* and recurrence of its infection. However, the underlying molecular mechanisms in these processes remain incompletely understood and warrant further investigation.

#### 3.2.4. Chemotaxis

Chemotaxis directs amoeba movement according to certain chemicals in their environment. This is important as *Acanthamoeba* moves towards the highest concentration of food molecules, or to flee from poisons. *Acanthamoeba* exhibits chemosensory responses as observed by their response to a variety of bacterial products or potential bacterial products by moving actively towards the attractant. *Acanthamoeba* responded to the chemotactic peptides formyl-methionyl-leucyl-phenylalanine, formyl-methionyl-leucyl-phenylalaninebenzylamide, lipopolysaccharide and lipid A. In addition, significant responses to cyclic AMP, lipoteichoic acid and N-acetyl-glucosamine were also found. Interestingly, chemotactic peptide antagonists, mannose, mannosylated bovine serum albumin and N-acetyl-muramic acid all yielded non-significant responses. Pretreatment of *Acanthamoeba* with chemotactic peptides, bacterial products and bacteria reduced the directional response to attractants. *Acanthamoeba* grown in the presence of bacteria appeared more responsive to chemotactic peptides. Treatment of *Acanthamoeba* with trypsin reduced the response of cells to chemotactic peptides, though sensitivity was restored within a couple of hours [7, 92]. These findings suggest that *Acanthamoeba* membranes possess receptors sensitive to these bacterial substances, which are different from the mannose-binding protein involved in binding to the host cells to produce cytotoxicity or involved in binding to bacteria during phagocytosis. The rate of movement is relatively constant (ca. 0.40 μm per sec), indicating that the locomotor response to these signals is a taxis, or possibly a klinokinesis, but not an orthokinesis [92].

#### 3.2.5. Ubiquity

*Acanthamoeba* has been found in diverse environments, from drinking water to distilled water wash bottles, deep ocean bottom and Antarctica. It is therefore not surprising that human beings encounter and interact regularly with these organisms, as is evidenced by findings that in some regions, up to 100% of the population tested possess *Acanthamoeba* antibodies, suggesting that these are one of the most ubiquitous protists and often come into contact with human beings, and given the opportunity (e.g. contact lens wear), can cause serious infections.

#### 3.2.6. Biofilms

Biofilms are known to play an important role in the pathogenesis of *Acanthamoeba* keratitis. Biofilms are microbially-derived sessile communities, which can be formed in aqueous environments as well as on any materials and medical devices including intravenous catheters, contact lenses, scleral buckles, suture material, and intraocular lenses. In the instance of contact lenses, biofilms are formed through contamination of the storage case. Once established, biofilms provide attractive niches for *Acanthamoeba* by fulfilling their nutritional requirements as well as providing resistance to disinfectants. In addition, this allows higher binding of *Acanthamoeba* to contact lenses. For example, *Acanthamoeba* exhibits significantly higher binding to used and *Pseudomonas* biofilm-coated hydrogel lenses compared to unworn contact lenses. The abundant nutrient provided by the biofilm encourages transformation of *Acanthamoeba* into the vegetative, infective trophozoite form, and it is important to remember that binding of *Acanthamoeba* to the human corneal epithelial cells most likely occurs during the trophozoite stage as cysts exhibit minimal binding. These findings suggest that biofilms play an important role in *Acanthamoeba* keratitis in wearers of contact lenses and preventing biofilm formation is perhaps an important preventative strategy [9, 118].

#### 3.2.7. Host factors

The factors that enable *Acanthamoeba* to produce disease are not limited solely to the pathogen, but most likely involve host determinants [17, 115]. Evidence for this comes from recent studies in the UK, Japan and New Zealand, which suggested that the storage cases of contact lenses of 400–800 per 10,000 asymptomatic wearers are contaminated with *Acanthamoeba*. This number is remarkably high compared with the incidence rate of *Acanthamoeba* keratitis in wearers of contact lenses, which is around 0.01–1.49 per 10,000 [46]. These findings suggest that factors such as host susceptibility, tissue specificity, tear factors, sIgA, corneal trauma, as well as environmental factors such as osmolarity may be important in initiating *Acanthamoeba* infections. However, the extent to which such host factors contribute to the outcome of *Acanthamoeba* keratitis is unclear because host factors are more complex and difficult to study than those of the pathogen. Overall, it can be concluded that *Acanthamoeba* traversal of biological barriers and to produce disease is a complex process that involves both pathogen as well as host factors.

## 4. *Acanthamoeba* keratitis treatment: a problem with no simple solution?

The treatment of *Acanthamoeba* keratitis has evolved since the first medical cure was reported in 1985 [46, 54, 86, 103–106, 111, 112]. Early diagnosis and aggressive medical therapy has improved the management of this difficult infection. Other reported factors that may facilitate effective medical therapy and an improved outcome include early epithelial debridement (to remove the majority of organisms) and penetrating keratoplasty in medically-resistant cases.

So far, no chemotherapeutic agent has been described as a single effective treatment against AK, regardless of the isolate or genotype that causes it. This is because there are many factors, including the wide range of virulence traits that different isolates possess, which makes it almost impossible to establish a correlation between *in vitro* and *in vivo* efficacies. Nevertheless, to establish the most effective treatment regimen is not easy for several reasons such as the relatively small number of reported cases of AK, variable pathogenicity of different strains, and the intrinsic fluctuating nature of the disease process.

### 4.1. Is there a single effective treatment against AK?

There are currently no methods or a single drug that can eliminate both cystic and trophozoite forms, while the trophozoite form is much more readily eliminated [46, 47, 54, 56].

The available reported treatment regimens in the literature have varied widely depending on the manifestation of the disease, the general health status of the infected cornea, and the experience of the clinician. For example, in the original AK case reported by Naginton et al. [reviewed in 46, 54], numerous topical antimicrobial preparations were tried in conjunction with steroids, but both eyes eventually required grafting. Recent years have brought us knowledge of more specific antimicrobials, although unfortunately, surgical grafting of the cornea remains the last solution in case of severe infection.

### 4.2. Current therapeutic approaches

Current AK treatment consists of topical antimicrobial agents, which can achieve high concentrations at the site of infection. Moreover, due to the existence of a cyst form in *Acanthamoeba* that is highly resistant to therapy, a combination of agents is generally used.

Most of the currently used topical agents are effective against trophozoites and cysts of *Acanthamoeba* such as biguanides, (i) PHMB [52, 54, 58], which is effective at low concentrations (0.02%), but is unfortunately toxic to human corneal cells [52, 54], and (ii) chlorhexidine, which is effective against both amoebic forms, and at minimal concentrations is not toxic to corneal epithelial cells [52, 54, 58, 86]. Chlorhexidine 0.02% is often used in combination with aromatic diamidines such as 0.1% propamidine isethionate Brolene^®^ (Sanofi, UK), 0.15% dibromopropamidine, hexamidine 0.1% Desomedine^©^ (Chauvin, France) and neomycin, showing good results if the treatment is applied early in the development of the infection [54, 86]. Unfortunately, propamidine and hexamidine are not available in all countries.

These topical antimicrobials are administered every hour immediately after corneal debridement or for the first several days of therapy. These agents are then continued hourly for 3 days (at least nine times/day is recommended) depending on clinical response. The frequency is then reduced to every 3 h. Two weeks may be required before a response is observed, and the total duration of therapy is a minimum of 3–4 weeks. Some authors also recommend treatment for 6–12 months. Moreover, when therapy is discontinued, close observation of the patient is suggested in order to avoid recurrent infection. Some patients have been successfully treated using an antiseptic as monotherapy; if this is attempted, it should be reserved for patients with early disease.

### 4.3. Biguanides as first line treatment against AK

PHMB and chlorhexidine have been reported to be the most effective drugs for treatment of infection and in combination they have been reported to be effective against both cysts and trophozoites [23, 52, 54, 79, 108]. Regarding these two drugs, it is important to mention that they are active against a wide spectrum of pathogens by increasing cytoplasmic membrane permeability. Chlorhexidine and PHMB both contain highly charged positive molecules capable of binding to the mucopolysaccharide plug of the ostiole resulting in penetration of the amoeba. The drug then binds to the phospholipid bilayer of the cell membrane which is negatively charged resulting in damage, cell lysis and death [52].

Among the observed side effects, toxic keratopathy may develop at any time, necessitating significant alteration in this treatment plan [46, 54, 98]. Elevated intraocular pressure as well as increased inflammation often requires the use of antiglaucoma medication and cycloplegics. The role of topical corticosteroids as well as surgical intervention with therapeutic penetrating keratoplasty in the management of this infection remains controversial and is discussed later.

Brolene^®^ may be accompanied by drug toxicity and resistance and topical treatment with miconazole can lead to epithelial toxicity [29, 54, 56]. According to Turner et al. [107], resistance is mainly due to the exocyst and endocyst, which forms a double-walled protective barrier to biocides. Cysts may also be resistant to biocides because they show little or no metabolic activity and because of selection pressure due to continuous drug exposure [46, 106, 107]. If resistance to drugs occurs, keratoplasty may be used [29, 46, 51, 54]. Ueki et al. [108] stated that recommended treatment for AK includes corneal scrape with antifungal drugs and antibiotic treatment. However, antifungal, antibacterial, antiviral and even corticosteroids used can complicate matters because they cause initial improvement then worsening of the disease [46, 54].

### 4.4. Steroids controversy in AK treatment

No clear consensus exists about use of steroids. Most authorities recommend that steroid use is probably best avoided. Patients receiving steroids should continue antiamoebic therapy for several weeks after the steroids are stopped. In the case of a persistent infection with inflammation, corticosteroids may be used. However, their use is controversial because they cause suppression of the immunological response of the patient. Moreover, corticosteroids produce inhibition of the processes of encystation and excystation of *Acanthamoeba*, which could be a cause of the appearance of resistance problems [103]. Recent studies have highlighted an association of topical corticosteroids and a diagnostic delay of AK. Moreover, there is some evidence that suggests that steroid use may result in increased pathogenicity of the amoebae [85]. McClellan et al. [63] demonstrated in an *in vivo* model that the addition of topical corticosteroids, even at low doses, promotes an increase in the number of trophozoites, produced by excystment in the infected corneal stroma. This exposes patients to the risk of significantly greater corneal destruction through an increase in amoebic load, which may be greater than the increased chemotherapy effect on trophozoites compared with the more resistant cysts [63, 85].

Furthermore, corneal transplantation (keratoplasty) is another therapeutic option when topical treatment has failed. This intervention is recommended if in the acute phase of infection, the cornea becomes too thin or has been damaged, and vision is limited [51, 69]. Nevertheless, there is a risk of not eliminating all the trophozoites or cysts that could colonize the new cornea [98]. A variation of keratoplasty called DALK (Deep Anterior Lamellar Keratoplasty) has been proposed to be more effective in increasing the survival of transplanted corneal cells and to prevent entry of pathogenic organisms at the time of surgery [72].

### 4.5. *In vitro* drug sensitivity testing and personalised treatments in AK patients

*In vitro* drug sensitivity testing, although rarely used, may be helpful in refractory cases. However, such testing has its limitations and may not be practical for the clinician. Not only may drug sensitivities be variable between strains, but a strain may also become resistant to formerly effective drugs. In addition, testing results may differ between laboratories and in some cases may not correlate with the clinical course. Despite these problems, drug sensitivity testing may offer the clinician a small edge in improving chances of therapeutic success and should be employed when possible. Recently, a patient suffering from severe AK was healed after a personalized treatment with voriconazole, when sensitivity to this drug was assayed after isolation of the amoebae from the patient’s cornea [5].

### 4.6. Surgical management of AK

Therapeutic penetrating keratoplasty was the mainstay of treatment for AK before the development of early diagnosis and aggressive medical therapy [10, 21, 36, 72]. The role and timing of penetrating keratoplasty in AK still remains poorly defined. Certainly pending or frank corneal perforation is a clear indication for surgical intervention. However, other indications for surgery are not well defined.

Therapeutic penetrating keratoplasty should be considered when the infectious process spreads to the paracentral corneal stroma despite maximum antiamoebic therapy [21]. Performing this procedure on a more localized infection may allow total removal of the organisms by excising the clinically involved tissue as well as a rim of clear surrounding cornea. This procedure allows the donor tissue to be placed into a relatively undamaged and hence non-immunocompromised recipient bed. After surgery, medical therapy should be continued for at least several months to help ensure elimination of any residual *Acanthamoeba* organisms in the recipient stromal tissue. Once the infection has spread into the peripheral cornea, however, the likelihood of achieving a surgical cure is markedly diminished. Intensive medical management is required for several months to eradicate the organism prior to keratoplasty. Unfortunately, the prognosis in these cases is poor and reinforces the rationale for earlier rather than later surgical treatment.

Recently, bipedicle conjunctival flap (CF) and cryopreserved amniotic membrane graft (AMG) have been reported to be effective in AK. They restore ocular surface integrity and provide metabolic and mechanical support for corneal healing. Nevertheless, in the case of large corneal perforation, penetrating keratoplasty to restore ocular integrity remains as the only effective surgical option [1].

### 4.7. Novel therapeutic approaches

Recently, the widespread use of photorefractive surgery has inspired its use in the setting of AK. Kandori et al. [44] reported four cases in 2010, where early stage AK was treated with standard topical therapy, but developed large corneal abscesses in the upper third thickness of the stroma. These were removed using laser phototherapeutic keratectomy (PTK); all eyes experienced no disease recurrence and final acuities ranged from 20/16 to 20/25.

Cross-linking is another relatively new treatment option that has been applied to AK. While *in vitro* studies by Kashiwabuchi et al. [45] and Del Buey et al. [25] have shown no amoebicidal effect of riboflavin combined with UVA exposure, clinical case reports have shown a much more promising picture. Garduño-Vieyra et al. [32] administered collagen cross-linking to a patient in Mexico instead of topical medical therapies, which were not commercially available. Significant improvement was observed after 24 h, with symptoms resolving within 3 months, and 20/20 vision was obtained after 5 months. Khan et al. [49] have since reported three similar cases which responded equally well to cross-linking, with all ulcers closing within 7 weeks. In subsequent PK surgery for scarring, no organisms were detected in excised tissue. It is possible that the collagen stabilizing effect prevents further tissue damage and isolates and prevents reproduction of the amoebae. Although individual case report results seem promising, there are no formal clinical trials thus far to recommend incorporation into standard practice.

### 4.8. In the search of novel drugs against AK

A new path may be the application of alkylphosphocholines. These are phosphocholines esterified to aliphatic alcohols. They exhibit *in vitro* and *in vivo* antineoplastic activity and have been shown to be cytotoxic against *Leishmania* spp., *Trypanosoma cruzi* and *Entamoeba histolytica*. A recent study has demonstrated that particularly hexadecylphosphocholine (miltefosine) is highly effective also against various strains of *Acanthamoeba*. Moreover, it has recently been applied in combination with PHMB in AK in Austria with successful outcomes [8, 78, 79].

Recently, the creation of a “pharmaceutical phylogeny” has been started for *Acanthamoeba* in order to elucidate and select new therapeutic targets [54, 58, 86]. The phylogeny of *Acanthamoeba* is closer evolutionarily to human beings than many other eukaryotic pathogens [69]. Therefore, part of the hypothesis is that many biochemical processes as well as therapeutic targets are evolutionarily conserved and are similar in related organisms. This implies that there will be a large number of processes, some still unknown, in *Acanthamoeba* that are similar to those of human beings. Therefore, treatments that affect the host could also affect the parasite, for example, phospholipid analogues as mentioned above which have been demonstrated to be effective against *Acanthamoeba* [2, 86]. However, even though many biological processes are similar in *Acanthamoeba* to other eukaryotic cells, some proteins such as tubulins are not sensitive to inhibitors that are normally used against them [86]. Therefore, it is also interesting to find those targets that are specific to *Acanthamoeba* in order to attack the parasite without affecting the host. These targets may be of a different nature: specific gene products, biological processes themselves, transcription or translation mechanisms or physical attributes such as cell membranes. In addition, many antibiotics active against *Acanthamoeba* have a mechanism of action which is still unknown. In recent years, the possibility to design and synthesize specific small interfering RNAs (siRNAs) for gene silencing have made RNAi techniques a powerful tool for the study and understanding of new cellular pathways of proteins whose functions are still unknown, as well as for their use as a therapy against various diseases [14, 88]. In the case of *Acanthamoeba*, siRNAs have been used successfully to identify potential therapeutic targets and even recently to establish a target and propose statins as a future therapy against *Acanthamoeba* strains [58].

Other drug targets that have been validated using the same approach, but without the elucidation of an available chemical alternative (active principle/drug) include glycogen phosphorylase and other cellulose synthesis related enzymes, serine proteases and myosin IC [4, 27, 53, 55, 57, 65]. The recently published *Acanthamoeba castellani* genome data will further assist in the development of novel therapeutics in the near future [19].

## 5. Concluding remarks

The number of reported cases of *Acanthamoeba* keratitis is increasing worldwide every year, due to increasing contact lens use for vision correction and cosmetic purposes. Increased awareness combined with early diagnosis of the disease is currently a good pathway towards better outcomes. However, knowledge about the pathogenesis and cellular differentiation processes in *Acanthamoeba* are still not fully known and urgently require further investigation. They hold the key to improved diagnosis and to development of effective therapeutic approaches.
